# Justifications for coercive care in child and adolescent psychiatry, a content analysis of medical documentation in Sweden

**DOI:** 10.1186/s12913-016-1310-0

**Published:** 2016-02-19

**Authors:** Veikko Pelto-Piri, Lars Kjellin, Christina Lindvall, Ingemar Engström

**Affiliations:** University Health Care Research Center, Faculty of Medicine and Health, Örebro University, SE-701 82 Örebro, Sweden; FPS, Mellringevägen 120 B, SE-703 52 Örebro, Sweden

**Keywords:** Psychiatry, Coercive care, Medical records, Decision making, Values, Ethics, Children, Adolescents, Content analysis, Sweden

## Abstract

**Background:**

There has been considerable interest in normative ethics regarding how and when coercive care can be justified. However, only a few empirical studies consider how professionals reason about ethical aspects when assessing the need for coercive care for adults, and even less concerning children and adolescents. The aim of this study was to examine and describe how professionals document their value arguments when considering the need for coercive psychiatric care of young people.

**Methods:**

All 16 clinics that admitted children or adolescents to coercive care during one year in Sweden were included in the study. These clinics had a total of 155 admissions of 142 patients over one year. Qualitative content analysis with a deductive approach was used to find different forms of justification for coercive care that was documented in the medical records, including Care Certificates.

**Results:**

The analysis of medical records revealed two main arguments used to justify coercive care in child and adolescent psychiatry: 1) *the protection argument* - the patients needed protection, mainly from themselves, and 2) *the treatment requirement argument* - coercive care was a necessary measure for administering treatment to the patient. Other arguments, namely the caregiver support argument, the clarification argument and the solidarity argument, were used primarily to support the two main arguments. These supportive arguments were mostly used when describing the current situation, not in the explicit argumentation for coercive care. The need for treatment was often only implicitly clarified and the type of care the patient needed was not specified. Few value arguments were used in the decision for coercive care; instead physicians often used their authority to convince others that treatment was necessary.

**Conclusions:**

One clinical implication of the study is that decisions about the use of coercive care should have a much stronger emphasis on ethical aspects. There is a need for an ethical legitimacy founded upon explicit ethical reasoning and after communication with the patient and family, which should be documented together with the decision to use coercive care.

## Background

It is generally regarded that coercion may be used in psychiatry if a person is believed by others to be in need of treatment for a serious mental illness and to pose a danger to themselves or others [1, 2]. There is an ethical challenge when a person that appears to be in need of psychiatric treatment, does not seek treatment or is actively refusing it. This conflict between autonomy of the patient on the one hand and the need for care and protection of persons on the other hand, is an example of a value conflict that arises for staff when considering coercive care for a patient. A study of the psychiatric legislation in the Nordic countries identified several values that may overlap as well as conflict such as, for example, respect for autonomy, integrity, beneficence, justice and sanctity of life [3]. Since the question of whether a patient is in need of coercive care is value-laden, the psychiatric assessment of the patient can not only be based on facts but has to be built on a combination of facts and values [4].

The concepts of *decision competence* and *best interest* are frequently used in ethical discussions about patients in psychiatry who do not comply with the suggested treatment [5, 6]. From an ethical standpoint, these issues become even more complicated when the patient is under 18 years of age. Parents have the primary obligation and responsibility for caring for and raising their child, but they are also entitled to help from society to cope with this task [7]. In the case of a child having serious psychiatric problems, a decision to use coercive care can, at least in Sweden, be made against the parents’ wishes [8]. The Swedish legislation has adapted to the Convention on the Rights of the Child [7]. Children, irrespective of age, have the right to participate in their healthcare to the extent that is possible with regard to maturity [7]. Participation in treatment decisions has been reported to be of therapeutic value and have positive effects on the self-confidence and self-esteem of adolescents [9, 10].

According to the Swedish Compulsory Mental Care Act [11], coercive care may only be given if the patient i) is suffering from a serious mental disturbance, and ii) has an absolute need of inpatient psychiatric care due to his/her mental state and general personal circumstances, and iii) objects to such care. The question of whether the patient, due to mental disturbance, is a danger to others should also be taken into consideration. A licensed physician in public health care is entitled to issue a Care Certificate if they examine a patient and find that the preconditions for coercive care are likely to be fulfilled. When the Care Certificate is issued, the patient is taken to a psychiatric clinical department at a public hospital where the decision regarding coercive care is made within 24 h by a psychiatrist after a new examination of the patient. In the present paper, we define the concept ‘coercive care’ as care and treatment given with legal authority according to the Compulsory Mental Care Act. The only regulation that is specific to children is that they are entitled to their own legal representation if they are above 15 years of age. The Act does not distinguish between children and adults when it comes to the criteria for coercive care, which differs, for example, from the Finnish Act in which coercive care may be used in order to secure the child’s health and development [12].

There has been a considerable interest in normative ethics about how and when coercive care can be justified [13–15]. However, only a few empirical studies consider how professionals reason about ethical aspects when assessing the need for coercive care, and these studies only pertain to adults. The decision on coercive care is not easy. A recent study reported that 45 % of the physicians found it difficult to use the two medicolegal criteria in Norwegian legislation: need of treatment and risk of danger to self or others [16]. In an interview study [17], it was found that a paternalistic perspective was dominant in the assessment. The decision was described as being in the patient’s best interest and the patients were described as lacking decision-making capacity. Two survey studies [18, 19] indicate that physicians also considered the severity of the patient’s disorder as well as the associated risks when making decisions about coercive care. They assessed the benefits of coercive care for a patient as opposed to the ethical costs of the violation of the patient’s autonomy [18]. Three studies found that physicians also took into consideration how their decision would affect others and sometimes they could be influenced by pressure from healthcare workers, family or the police [16, 19, 20].

With regard to adolescents, it has been reported that psychiatrists in Finland are of the opinion that the criteria for coercive care of minors should be broader than for adults and that coercive care should also be used as a preventive measure [21]. Two studies of medical records indicate that coercive care was associated with psychotic symptoms, mental retardation, temper tantrums, substance abuse, violent behaviour, and suicide risk [22, 23]. In an earlier study from our research group [24], staff members in child and adolescent psychiatry were asked to describe work situations that contained some sort of ethical consideration. We found six arguments that were used to ethically justify decisions about coercive care:

1. *The protection argument.* People lacking the ability to take responsibility for themselves must be protected by society through coercive care. This concept is often referred to as weak paternalism and is seen as imperative and ethically justified when the situation is obvious; e.g. in suicide attempts or overt violence.
2. *The solidarity argument.* The welfare society has a wide obligation to ensure the wellbeing of its citizens. This obligation justifies the use of coercion against people who have the ability to take responsibility but are seen as making unreasonable choices that may jeopardize their health. This is often referred to as strong paternalism and can be used to justify coercion; e.g. to prevent violent crime in society.
3. *The treatment requirement argument.* Coercive care creates the necessary prerequisites for the treatment that the patient, according to staff, is in great need of. Coercion is seen as the only possible option for controlling the illness in certain cases where all other treatment options have been exhausted. The intention is to restore the patient’s mental functioning, thereby increasing his/her capacity to take (other) autonomous decisions.
4. *The clarification argument.* The decision on coercive care clarifies for staff members that they are entitled to use coercive measures when they have assessed a treatment need and the patient refuses to participate. It is reassuring for staff and also makes the conditions of care prerequisites clear for patients and parents alike.
5. *The parent support argument.* If parents are not able to fulfil their parental role coercive care is justified in order to strengthen or complement parental authority. It can also be justified if the parents are unwilling or unable to participate in the care.
6. *The everyday care argument*. Decision on coercive care makes it easier for staff in their everyday work to provide adequate care, making it easier to avoid hassles and troubles in everyday care.

In summary, there are some studies in child and adolescent psychiatry that consider how staff reason about coercive care in principle and how it may be justified, but we have not found any studies about arguments for coercive care expressed by staff in medical records. We did not expect to find any extensive ethical reasoning in the medical records, especially not in the Care Certificates, since these are legal documents. However, we did expect that relevant facts would be presented in such a way that arguments for coercive care could be extrapolated, and that most of these arguments would contain not only facts but also values. Since coercion is ethically challenging, the arguments used in in these assessments of young patients are, therefore, of great importance.

The aim of this study was to examine and describe how professionals express and document their value arguments when considering the need for coercive psychiatric care of young people.

## Methods

### Setting and material

We asked all 21 child and adolescent psychiatric clinics in Sweden that were able to provide coercive care according to the Compulsory Mental Care Act [11] to send us anonymised (names and other identifying information blacked out) paper copies of the complete medical records of patients treated under the aforementioned Mental Care Act during one year (July 2002 – June 2003). Of these 21 clinics, 16 used coercive care during that year. These clinics admitted children and adolescents under 18 years of age who were assessed to be in need of psychiatric inpatient care from the respective surrounding catchment area, and 143 patients were involuntarily admitted 156 times. One patient with one admission was excluded, since the patient was sentenced to forensic psychiatric care.

Only public hospitals have the right to use coercive care in Sweden. We were granted permission by the Regional Research Ethics Committees in Sweden to access the complete medical records for research purposes. After several reminders, all 16 clinics in question sent us the document we requested. The medical records were often extensive and the documents were not well organised. A central part of this documentation with regard to the research question was the Swedish Care Certificate; a four-page form with pre-defined questions concerning the patients’ medical and social background, the current psychiatric condition including the circumstances that necessitates coercive care, somatic and mental status, and a final conclusion and decision about coercive measures that are found to be needed.

### Study population

We had access to the medical records from all the 155 admissions but only 143 Care Certificates. The 142 patients in the study were almost exclusively adolescents. Median age was 16 years and 89 % of patients were aged 14–18, only 15 patients were 10–13 years old. There were about twice as many girls as boys. The length of stay in inpatient care was on average 43 days per patient and year. The patients’ problems varied considerably, but the most frequent diagnoses were eating disorders, psychoses, depression with or without suicide attempts, and neuropsychiatric disorders. Twenty-one per cent suffered from concurrent substance abuse and nine per cent were asylum seekers (Table 1).

**Table 1:** Background data of the study population (*n* = 142)

Gender, n (%)	Girl	91 (64.1)
Age, Min/Md/Max^a)^		10/16/18
Days in hospital, Min/Md/Max^a)^		0/14/679
Main ICD-10 diagnosis, n (%)	Mental and behavioural disorders due to psychoactive substance use, F10-19	6 (4.2)
	Schizophrenia, schizotypal and delusional disorders, F20-29	12 (8.5)
	Mood (affective) disorders, F30-39	34 (23.9)
	Neurotic, stress-related and somatoform disorders, F40-48	27 (19.0)
	Behavioural syndromes associated with physiological disturbances and physical factors, F50-59	19 (13.4)
	Disorders of adult personality and behaviour, F60-69	4 (2.8)
	Pervasive and specific developmental disorders F80-F89	7 (4.9)
	Behavioural and emotional disorders with onset usually occurring in childhood and adolescence, F90-98	15 (10.6)
	Missing diagnoses, uncertainty in assessment or an unspecified mental disorder, F99	9 (6.3)
	Intentional self-harm, X	4 (2.8)
	Observation, Z	5 (3.5)
Substance abuse, n (%)		30 (21.1)
Asylum seekers, n (%)		13 (9.2)

^a^Minimum/Median/Maximum

### Analysis and interpretation

We used qualitative content analysis [25, 26] in order to find the different forms of justifications for coercive care that formed the basis for the decisions; as documented in the medical records and especially in the Care Certificates. In qualitative content analysis, the researcher creates categories or themes based on the research question. This method is suitable when the researcher wants to provide a good description of a large amount of qualitative material [27]. Our main focus was on the day when the Care Certificate was written, the 24 h after that, and the week prior to that day. A summary was made consisting of the patient’s background, current situation, the psychiatric assessment and justification of coercion (Table 2).

**Table 2:** An example of summary of data from one case with coding; (1) the protection argument, (2) the solidarity argument, (3) the treatment requirement argument, (4) the clarification argument, (5) the parent support argument and (6) the everyday care argument

	Background	Current situation	The psychiatric assessment	Justification of coercion
Care Certificate	The parents have been trying to control the patient’s eating habits, but have not been successful (5). She has had previous contact with psychiatric services, but her eating behaviour is unchanged. Has previously been admitted voluntarily (BMI 14). The patient’s older sister has anorexia.	During the spring, the patient has dramatically reduced in weight; lost 7 kg since April.	Severe anorexia nervosa. The patient acts by screaming, crying and locking herself in the bathroom. She refuses treatment and tube feeding (3). She has anorexic thoughts and delusions about body image (1).	Substantial risk to the patient’s life due to self-starvation (1). Lack of insight (3).
Other document-ation^a^	Lives with her two parents and an older sister. She has two older siblings who have moved away from home.	The parents sought acute care with the patient; she was on the waiting list for treatment in Anorexia-Bulimia Clinic. The patient refuses tube feeding and totally refuses to eat (3). Care Certificate written due to severe self-starvation (1).	The patient has rapidly lost weight (1). Experiencing strong anxiety about feeding and tube feeding.	

^a^Medical records, except for the Care Certificates

In the analysis, we were looking for value arguments used to justify coercive care by using a deductive approach [26], since we had earlier results from a pilot study [24]. The six arguments presented earlier [24] were used as predefined categories (for an example of coding, see Table 2). A deductive approach entails only that the start of the analysis should be based on predefined categories. It was important for us to pay attention to data that might contradict or modify the pre-defined categories or that data might consist of other important categorises besides the predefined. CL made an initial coding, VP went through this coding with more stringent criteria. LK conducted an inter-rater assessment by randomly selecting 36 cases, reading and commenting on them. In most cases, LK agreed with the coding of VP. Problematic coding occurred in particular regarding the parent support argument. The problematic cases were discussed with all authors until agreement was reached, and all cases were then reviewed to achieve a consistent categorisation throughout. In the analysis of the protection argument, VP, supported by LK, searched for cases that documented dangerousness to self or to others. This was divided into three categories: mortal danger, risk of harm and absence of documentation about risk (Table 3).

**Table 3:** Categorisation of the documented assessments of patients’ dangerousness towards themselves and others

Risk	To the patient	To others
Mortal danger	Positive assessment of risk of suicide and/or the patient had attempted suicide (including suicide threats).	Positive assessment of risk of mortal danger to others and/or the patient had recently made an assassination attempt (including making explicit death threats) or committed serious assault.
Risk of harm	Positive assessment of risk of self-harm or the patient had recently self-injured.	Positive assessment of harm to other person/s and/or the patient had recently harmed somebody else.
Absence of documentation	No documentation.	No documentation.

### Ethics

The patients that were included were not informed about the study. Contacting each individual to ask for consent to use medical records regarding their involuntary psychiatric care was regarded by the Research Ethics Committees as ethically problematic since reminders of past negative events may cause distress. It would also be difficult on a practical level and would probably result in a large number of dropouts. The study was approved as a multicentre study by the Research Ethics Committee in Orebro (reg. 411–02).

## Results

### Arguments used to justify coercive care

The overall result, according to our interpretation, was that the main arguments used to justify coercive care were the protection and the treatment requirement arguments (Table 4). The other arguments were mainly used to support these two arguments and were mostly used when describing the current situation, but not in the explicit argumentation for justifying coercive care, and they were less common in in the Care Certificates than in the rest of the medical records.

**Table 4:** Arguments used in decisions of coercive care

Arguments	Psychiatric Care Certificate *n* = 143 (%)	Other Documentation^a^ *n* = 155 (%)	Complete medical records *n* = 155 (%)
The protection argument	137 (96)	134 (86)	153 (99)
The treatment requirement argument	80 (56)	70 (45)	107 (69)
The caregiver support argument	24 (24)	59 (37)	75 (48)
The clarification argument	18 (13)	18 (12)	33 (21)
The solidarity argument	1 (1)	5 (3)	6 (4)
The everyday care argument	0 (0)	0 (0)	0 (0)

^**a**^Medical records, except for the Care Certificates

### Main arguments

*The protection argument* was used in 153 out of 155 cases (Table 4) and all 155 cases had some documentation of danger for the patient or others (Table 5). In 117 cases out of 155, there was a documented risk of suicide, or a recent attempt, or another life-threatening situation such as dehydration due to an eating disorder. Some of these patients had symptoms such as hallucinations or compulsive behaviours that could result in serious injury to the patient or others. In seven cases, there was no documentation about the patients being a danger to themselves, and for the majority of patients there was no documentation about dangerousness to others (Table 5).

**Table 5:** Assessments of patients’ dangerousness towards themselves and others

	Mortal danger to others	Risk of harm to others	Not dangerous to others^a^	SUM
Risk of suicide	7	31	79	117
Risk of self-harm	4	17	10	31
Not dangerous to themselves^a^	1	6		7
SUM	12	54	89	155

^a^ = absence of documentation about assessments of patients’ dangerousness

There were only 12 cases with documentation about mortal danger to another person such as a recent assassination attempt, a serious beating that had been committed, or death threats that had been expressed. Nearly all of these threats were against the mother or other relatives. Sometimes the need for staff protection was mentioned. In several cases, the staff mentioned that the interior fittings needed to be protected from the patient. In summary, the Care Certificates portrayed patients in need of coercive care as persons who are not capable to take care of themselves and therefore must be protected from themselves.

*The treatment requirement argument* was used in 107 out of 155 cases, but in many other cases the need for care was only implied in the Care Certificates with a description of a severe psychiatric condition. Some usual arguments were that the patient was in need of a structured environment, needed to be under surveillance, or required a better assessment, and that coercive care was a prerequisite for interventions that the patient was in great need of. It was often mentioned that the patient refused voluntary treatment. However, the documentation seldom gave any descriptions as to what specific care was found to be necessary for the patient’s rehabilitation. In a few cases, it was documented that nutrition by tube was absolutely necessary to save the life of a starving patient. In other cases, the patient had neglected their medical treatment or it was merely stated that forced medication was necessary. After a new examination of the patient by a psychiatrist, 33 patients (21 %) were not considered to be in need of coercive care after 24 h. In two of these 33 cases, we found no documented psychiatric diagnosis or suspicion of serious mental illness. One decision was based on an urgent situation where a patient had expressed threats against his classmates, and in the other case the patient had acute alcohol intoxication.

### Supportive arguments

*The caregiver support argument* was used in 75 out of 155 cases and was often included as an explanation as to why the youth was taken to the psychiatric services. As a result of our analysis, we changed the name of this argument from the parent support argument to the caregiver support argument. As well as 59 families, there were also 16 special residential homes for adolescents that had great difficulties in handling adolescents who, according to documentation, were assessed as being in need of the support that an admission to inpatient care could give. Adolescents admitted from special residential homes often showed signs of a high suicide risk, frequently threatening or behaving violent towards staff, as well as being escape-prone. Many family members were completely exhausted after taking care of their sick child. There were also many parents who found it difficult to cope with parenting in general, due to substance abuse, mental illness or social problems. In one case, the staff did not manage to make contact with the parents at all before writing the Care Certificate.

*The clarification argument* was found in 33 out of 155 cases. This could indicate that staff had plans to use coercive measures and that a decision on coercive care made it clear to staff that these actions could be taken. In a few cases, a decision was documented that the patient was going to be subject to coercive treatment regardless of the patient’s or the parents’ wishes and they were clearly informed of this; such as when a patient who suffered from anorexia was told that she would be fed by tube with or without the parents or patient’s consent.

*The solidarity argument* could be found in six cases out of 155. In some cases, the psychiatric clinic took more responsibility than might be expected of them. We found documentation about adolescents who were going to be admitted to special residential homes and therefore stayed on the ward until they could be sent there. In other cases, the focus was on emergency situations where the psychiatric clinic took a responsibility that could have been taken by the social authorities. These cases were, according to the documentation, necessary interventions in emergency situations due to lack of other alternatives.

*The everyday care argument* was not found clearly stated in the documentation as an argument. However, it was clear that parents or staff at the residential homes could not succeed in getting the everyday care of the adolescent to function in a satisfactory manner. Members of staff were often afraid of the adolescent and wanted to get her/him admitted to psychiatric inpatient care.

## Discussion

The main finding in this study of medical records was that, in their assessment of the need for coercive care of adolescents, professionals used most frequently arguments about the need to protect the patient and to give necessary treatment. These arguments were used even more in the Care Certificates. A large majority of patients were described as suicidal, but the argument for risk of harm to others was rarely used. Physicians may be of the opinion that the right to treatment is a fundamental value [21], but despite this they rarely described what kind of treatment they considered to be necessary. A connected finding in our previous research is that some young people reported that they did not perceive receiving any special treatment during their stay as inpatients [10].

According to the Compulsory Mental Care Act, the physician should describe in the Care Certificate the ways in which other forms of treatment have been exhausted and why coercive care is considered to be the only available option left. No physician, however, was found to argue in such a way. Instead, they often used their authority as a physician and just stated that “coercive care is necessary”. This gives the impression that the decision was easy to make, which is in contrast to a study where many physicians reported having difficulties in using the two medicolegal criteria [16]. The Care Certificate is likely to be more readily accepted by authorities if it refers to a possible suicide risk [17], which may lead to an emphasis on risk of suicide or self-harm in relation to other relevant information about the patient. A paternalistic impression was given through the emphasis on lack of decision-making competence and on it being in the patient’s best interest to be admitted to coercive care. Little consideration was given to other solutions or to problematisation of the decision, which is consistent with a recent study [17] where the conclusion was that the decision of coercive care is most often taken from a paternalistic perspective.

There were large differences between Care Certificates and other documentation in the arguments used, which indicates that the physicians adapted the Care Certificates primarily to fulfil the legal requirements, which could be viewed upon as being of a paternalistic nature. In many cases, for instance, professionals wrote in other documentation about the need to support the parents in their parenting but they did not mention it in the Care Certificates since the legislation does not take parenting into account. The solidarity argument, which does not have any legal support, was rarely used in the medical documentation, even though staff often used it in general terms during interviews [24].

We did not expect to find any extensive ethical analysis in the Care Certificates since these acute situations often require quick handling, but we had not expected that it would be so hard to find value arguments about the need for coercive care. To determine whether a patient has a serious mental disorder or not involves value-laden considerations [28]. The question about the patient’s mental state and general personal circumstances is an even more value-based assessment [4]. It would appear that physicians in this study had, in many instances, reduced this legal requirement to just one issue, about whether the patient’s behaviour was a danger to themselves or others. Two patients did not have any documented serious mental disorder at all. The first one was in need of help in an emergency situation and the other one was considered to be dangerous to others. In these cases, the physician decided to write a Care Certificate anyhow. Emergency referrals are more likely to recommend coercion [29]. It seems that physicians in the Care Certificates are more likely to accept coercive care of young patients without any other argument than to protect the young patients, in comparison to the coercive care of adults where psychiatrists in interviews expressed more ambivalence about accepting danger to the patient as the only argument for coercive care [30]. These two decisions above were, as far as we understand, of an ethical nature but even in these cases, there were no statements that could be interpreted as an ethical reasoning to justify these decisions.

The methodical strength of this study is that we examined a complete annual population of patients. One weakness is that medical records are extensive documents, but very brief when it comes to clarifying the argumentation for coercive care and we could not ask questions to the physicians and other staff about their practical and ethical reasoning. Another weakness is that the categories were quite wide and partly overlapped.

## Conclusions

One clinical implication that can be drawn from this study is that an ethical turn, which implies focusing not only on diagnostic or legal but also on ethical aspects, is needed within the area of coercive care in child and adolescent psychiatry. We believe that decisions about the use of coercive care necessarily form part of the psychiatric realm but propose that there should be a much stronger emphasis on ethical aspects. When executing this power, it is essential that the system secures legitimacy both from society and from the family concerned. This needs to be taken into account and assumes that a communicative process, in which all the affected parties are included in the deliberations, is essential [4, 17]. Decisions should, therefore, be made only after a dialogue with all parties concerned, despite, or rather because of, the fact that the communication process in these situations is often complicated to handle [9, 31]. This legitimacy cannot be acquired by satisfying diagnostic and legal requirements alone. Subsequently, there is a need for an ethical legitimacy founded upon explicit ethical reasoning, which should be documented together with the decision for using coercive care.
